# Diagnostic parameters of modified two-tier testing in European patients with early Lyme disease

**DOI:** 10.1007/s10096-020-03946-0

**Published:** 2020-07-06

**Authors:** ME Baarsma, JFP Schellekens, BC Meijer, AH Brandenburg, T. Souilljee, A Hofhuis, JW Hovius, AP van Dam

**Affiliations:** 1 Amsterdam UMC, University of Amsterdam, Center for Experimental and Molecular Medicine, Amsterdam Infection & Immunity, Meibergdreef 9, Amsterdam, 1105 AZ the Netherlands; 2 Certe Laboratory of Infectious Diseases, Groningen, the Netherlands; 3 Centre for Infectious Diseases Research, Diagnostics and Laboratory Surveillance, National Institute of Public Health and the Environment (RIVM), Bilthoven, the Netherlands; 4 Izore, Centrum Infectieziekten Friesland, Leeuwarden, the Netherlands; 5 Epidemiology and Surveillance Unit, Centre for Infectious Disease Control, National Institute of Public Health and the Environment (RIVM), Bilthoven, the Netherlands; 6 Department of Medical Microbiology, OLVG, Amsterdam, the Netherlands; 7 Amsterdam UMC, University of Amsterdam, Department of Medical Microbiology, Amsterdam, Netherlands

**Keywords:** Lyme disease, Borreliosis, Serology, Modified two-tier testing, C6

## Abstract

Modified two-tier testing (MTTT) for Lyme borreliosis (i.e. confirmation with an EIA instead of an immunoblot) has been shown to have improved sensitivity compared with standard two-tier testing (STTT) in samples from American patients, without losing specificity. The current study assesses the sensitivity and specificity of various algorithms of MTTT in European patients with erythema migrans (EM) as a model disease for early Lyme borreliosis, and in appropriate controls. Four different immunoassays were used in the first tier, followed by either an immunoblot or the C6-EIA, or were used as standalone single-tier test. These tests were performed on consecutively collected sera of 228 Dutch patients with physician-diagnosed EM in the setting of general practice, 231 controls from the general population, and 50 controls with potentially cross-reactive antibodies. All the variants of MTTT that were studied had significantly higher sensitivity compared with their equivalent STTT, while retaining comparable specificity. Within the MTTT algorithms, classifying equivocal results as positive yielded better diagnostic parameters than classifying equivocal results as negative. The best diagnostic parameters were found using the Enzygnost-2 assay in the first tier, followed by a C6-ELISA in the second tier (sensitivity 77.6%, 95% CI 71.7–82.9; specificity 96.1%, 95% CI 92.7–98.2). This algorithm performed significantly better than the equivalent STTT algorithm in terms of sensitivity (*p* < 0.001), while maintaining comparable specificity (population controls *p* = 0.617). Our results show that MTTT can be a useful tool for the serodiagnosis of European patients with early Lyme borreliosis.

**Electronic supplementary material:**

The online version of this article (10.1007/s10096-020-03946-0) contains supplementary material, which is available to authorized users.

## Introduction

While a thorough clinical assessment is a physician’s foremost tool for diagnosing Lyme disease (Lyme borreliosis, LB), laboratory work-up is oftentimes required. The cornerstone of this laboratory work-up is serology. Traditionally, guidelines in both the USA and Europe have advised to perform serodiagnosis of LB by applying two-tier testing [1–3]. In the first tier, a highly sensitive (but possibly false-positive) enzyme immunoassay (EIA) is performed, after which positive or equivocal results require confirmation in a second tier by IgM/IgG immunoblotting. While some have come close, no single test has to the best of our knowledge attained or surpassed the combined sensitivity and specificity of standard two-tiered testing (STTT).

An important component of several EIAs is the VlsE protein. VlsE is highly immunogenic and antibodies are produced early after onset of infection with *Borrelia burgdorferi* sensu lato (Bbsl) [4, 5]. Within the VlsE protein, a 26-amino acid sequence named invariable region 6 (IR6) has been shown to be highly conserved among the various subspecies of Bbsl, and to be highly immunogenic [6]. This had led to development of the C6-ELISA in which a synthetic peptide based on IR6 is used as antigen. The C6-ELISA is used both in the USA and Europe [7, 8].

For the second tier, American healthcare providers rely on immunoblots prepared from native cultivated *B. burgdorferi* sensu stricto (Bbss) bacteria, which is the primary causative agent in North America. In Europe, LB is caused by a variety of Bbsl species (e.g., *B. garinii* (Bgar) or *B. afzelii* (Bafz)) [9]. It is, therefore, virtually impossible to standardize immunoblotting in Europe using *Borrelia* lysates with respect to choice of antigen and uniform interpretation criteria. As a result, European immunoblots rely primarily on recombinant antigens of the various subspecies of Bbsl prevalent on that continent [10]. Other drawbacks of immunoblotting are that it is considered laborious and may be prone to inter-assay variation as it is non-quantitative.

A further argument for revisiting the STTT algorithm is its limited sensitivity early in the course of the disease, specifically for diagnosing early localized LB (an erythema migrans, EM). A meta-analysis found that STTT has a sensitivity of approximately 50% in patients with an EM [11]. For this reason, most guidelines consider EM a clinical diagnosis and recommend against performing serological testing for early localized LB [1–3], even though extra certainty in the form of laboratory testing can be desirable, for instance when cutaneous lesions are atypical or vague.

Various solutions to the aforementioned problems with confirmatory testing by immunoblot have been proposed. In an American study comparing the diagnostic parameters of STTT vs. the C6-ELISA alone, the C6 assay was shown to be significantly more sensitive than standard two-tiered testing in EM, and comparably sensitive in various forms of disseminated LB [12]. However, this did come at the cost of a significantly lower specificity, even though the difference was small (C6 98.8%; STTT 99.5%) [12]. Other studies have examined the diagnostic parameters of so-called modified two-tier testing (MTTT) using different EIAs or ELISAs in both tiers [7, 13–15]. These studies showed that the MTTT algorithm had far better sensitivity in early LB than STTT, but without the associated loss of specificity. These findings—and others—have led the FDA to recently approve a *Borrelia*-EIA for use as confirmatory 2nd-tier test [16]. Previous research on MTTT has, however, been limited to sera from the USA. The differences in genospecies and diagnostic tests between Europe and the USA necessitate that these findings be reproduced in European sera, before MTTT algorithms can also be used on that continent.

In this multiple-gate case-control study, we have investigated the sensitivity of various assays and algorithms of MTTT in sera of European (Dutch) patients with physician-diagnosed EM as the most prevalent manifestation of early LB in Europe, and their specificity in a variety of control sera. We have used one of several commercial EIAs in the first tier, followed by a European immunoblot or the C6-ELISA in the second tier. Similar to findings from the USA, we hypothesize that MTTT will improve sensitivity of serological testing in early LB over STTT, while maintaining adequate specificity.

## Materials and methods

### EM sera

**Fig. 1:**
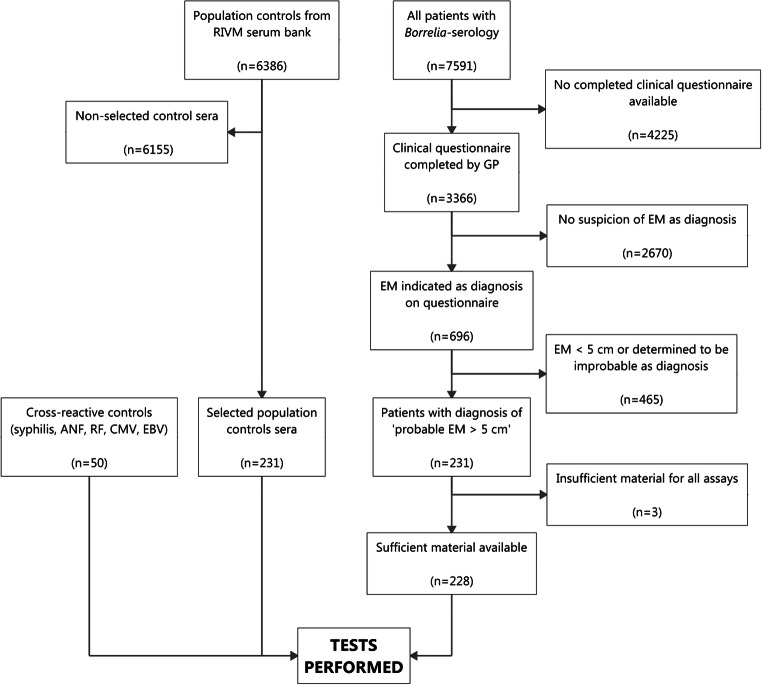
Serum selection

Sera from 228 patients with early localized LB (an EM) were used as cases. These were selected from all sera sent between October 2010 and October 2011 by general practitioners (GPs) requesting *Borrelia* serology at the Certe Laboratory of Infectious Diseases (Certe LID) in Groningen, the Netherlands. Clinical data were collected from GPs by questionnaire, which were sent to the GP before serological test results were available. Selection of participants was consecutive: the study was performed on sera from all patients (1) who had sera sent in during the given timeframe, (2) for whom the necessary clinical information was available, (3) who had a probable EM > 5 cm as diagnosis, and (4) for whom enough serum was available to perform all tests. All other sera were excluded. The EM diagnosis was made by the GP, based on Dutch guidelines [17]. It was made purely based on these clinical findings, and hence independently from serological test results. Sera were drawn at the time of the clinical diagnosis. The process of serum selection is also given in Fig. 1. Of the selected EM sera, 46.1% were collected from males and 53.9% from females. The median age of patients was 53 years (range 1 to 86, IQR 39–62 years). The aforementioned sample size was chosen independent from a sample size calculation.

### Population control sera

Two hundred thirty-one sera were selected from the serum bank archived at the National Institute of Public Health and the Environment of the Netherlands (RIVM) and were used as population controls (PopC) (Fig. 1) [18]. These sera were collected between 2006 and 2007 as a representative sample of the general population of the Netherlands. PopC sera were selected to yield an age and sex distribution comparable with the EM patients. Of the selected control sera, 45.9% were collected from males and 54.1% from females. The median age of population controls was 53 years (range 1 to 79, IQR 40–62 years).

### Cross-reactive sera

Ten sera with VDRL ≥ 1:32 and positive TPPA (syphilis), 10 sera with positive anti-nuclear factor (ANF), 10 sera with positive rheumatoid factor (RF), 10 sera positive for IgM and IgG antibodies to CMV, and 10 sera positive for IgM and IgG antibodies to EBV were used as cross-reactive controls (CRC) (Fig. 1). These sera were selected from the serum bank of Certe LID.

### Assays

Four commercially available immunoassays were performed on all samples (Table 1) in a non-blinded fashion. All sera which gave an equivocal or positive result in at least one of the EIAs/CLIA were tested by immunoblot. All assays were performed according to the manufacturers’ instructions. The Enzygnost-2 and immunoblot on EM sera were performed as part of normal clinical routine. All other assays were performed in batches, as were the Enzygnost-2 and immunoblot on PopC and CRC sera. In the interim, sera were stored at − 80 °C. An effort was made to keep thaw-refreeze effects to a minimum.

**Table 1:** Overview of assays

First tier				Second tier			
Abbr.	Name and manufacturer	Antibody	Antigen	Abbr.	Name and manufacturer	Antibody	Antigen
Enz1	Enzygnost borreliosis (Siemens, Eschborn, Germany)	IgM	Detergent extract of Bafz PKo	RecomL (STTT)	RecomLine Borrelia (Mikrogen, Neuried, Germany)	IgM	15 rec. proteins of Bbss, Bafz Bbav, Bspiel and Bgar
		IgG	*Identical*				
Enz2	Enzygnost borreliosis Lyme Link (Siemens, Eschborn, Germany)	IgM	Detergent extract of Bafz PKo				
		IgG	*Identical*, with added mix of 3 rec. VlsE variants			IgG	15 rec. proteins of Bbss, Bafz, Bbav, Bspiel and Bgar
Lia	Liaison B. burgdorferi (DiaSorin, Saluggia, Italy)	IgM	rec. OspC of Bafz PKo and rec. VlsE of Bbav PBi				
		IgG	rec. VlsE of Bbav PBi				
C6	C6 Lyme ELISA (Immunetics, Boston, USA)	IgM/IgG	rec. VlsE (IR6) of Bbss	C6 (MTTT)	C6 Lyme ELISA (Immunetics, Boston, USA)	IgM/IgG	rec. VlsE (IR6) of Bbss

*Bafz*, *B. afzelii*; *Bbav*, *B. bavariensis*; *Bgar*, *B. garinii*; *Bbss*, *B. burgdorferi* sensu stricto; *Bspiel*, *B. spielmanii*; *ELISA*, enzyme-linked immunosorbent assay; *OspC*, outer-surface protein C; *rec.*, recombinant; *VlsE*, variable major protein-like sequence, expressed

Assays were interpreted using cutoffs pre-defined by the manufacturer. A test was deemed positive when either the IgM component, the IgG component, or both were positive. A test was considered negative when neither component was reactive. A test was considered equivocal when both components were equivocal, or when one component was equivocal and the other negative.

### Algorithms

Three serodiagnostic algorithms were evaluated:

1. Single-tier: one of the EIAs/CLIA as sole test. Equivocal test results were classified as negative.
2. STTT: one of the EIAs/CLIA followed by immunoblot. This algorithm was evaluated in its standard form, i.e. equivocal test results in the EIAs were classified as positive, but equivocal test results in the immunoblot were classified as negative.
3. MTTT: one of the EIAs/CLIA (C6 excepted) followed by the C6-ELISA. This algorithm was evaluated both (3a) classifying equivocal test results as negative (‘strict’) and (3b) classifying equivocal test results as positive (‘permissive’).

### Statistical analysis

Diagnostic parameters of tests within each serum group were compared with one another using the McNemar test, or the exact McNemar where applicable. The 95% confidence intervals of proportions (i.e. of the sensitivity and specificity) were determined using Clopper-Pearson. The differences in sensitivity and specificity of the permissive vs. strict variant of MTTT were assessed using a one-sample *t* test, as algorithm interpretation rules prevented the McNemar test from being used in these comparisons. The same applied to the comparison of the MTTT-strict algorithm vs. the C6-ELISA as single-tier test, and the comparison of MTTT-permissive vs. the MTTT-permissive IgG only. We performed subanalyses to assess consistency of reactivity across the various conditions in CRC sera. For all analyses, *p* values < 0.05 were considered statistically significant.

### Ethical statement

The study was conducted according to the principles of the Declaration of Helsinki and in conformity with institutional regulations and guidelines. The study utilized only patient materials left over from standard clinical practice. Therefore, the Dutch Medical Research Involving Human Subjects Act does not apply to this study and no informed consent was asked from participants.

## Results

Using the single-tier algorithm, three assays performed comparably with regard to sensitivity (78–81%), except for the Liaison which showed a significantly lower sensitivity (70%) than all other assays (*p* < 0.01) (Table 2). All single-tier assays performed comparably with respect to specificity, both in PopC (89–94%) and CRC sera (62–78%) (Table 3). False positivity in CRC sera was mainly due to IgM cross-reactivity in patients with acute EBV or CMV infection (data not shown).

**Table 2:** Sensitivity of all algorithms

	Single-tier			STTT			MTTT-strict		MTTT-permissive	
	No. of true positive (sensitivity %)	95%-CI		No. of true positive (sensitivity %)	95%-CI		No. of true positive (sensitivity %)	95%-CI	No. of true positive (sensitivity %)	95%-CI
EM sera (*n* = 228)										
Enz1	180 (78.9)^1^	73.1–84.1	Enz1/RecomL	106 (46.5)	39.9–53.2	Enz1/C6	162 (71.1)^1,2^	64.7–76.8	173 (75.9)^1,4,5^	69.8–81.3
Enz2	184 (80.7)^1^	75.0–85.6	Enz2/RecomL	106 (46.5)	39.9–53.2	Enz2/C6	166 (72.8)^1,2^	66.5–78.5	177 (77.6)^1,4,5^	71.7–82.9
Lia	160 (70.2)^1^	63.8–76.0	Lia/RecomL	101 (44.3)	37.7–51.0	Lia/C6	153 (67.1)^1,2^	60.6–73.2	166 (72.8)^1,3,4,5^	66.5–78.5
C6	178 (78.1)*	72.1–83.3	C6/RecomL	102 (44.7)	38.2–51.4					

^1^*p* < 0.01 as compared with equivalent STTT using (McNemar/exact McNemar) | (*C6 vs. C6/RecomL *p* = 0.016)

^2^*p* < 0.01 as compared with single-tier C6-ELISA (one-sample *t* test)

^3^*p* < 0.01 as compared with single-tier C6 (exact McNemar)

^4^*p* < 0.01 as compared with equivalent MTTT-strict (one-sample *t* test)

^5^Not significant as compared with equivalent single-tier (exact McNemar)

A test result was considered to be (true) positive when either the IgM component, IgG component or both were positive

*95%-CI*, 95% confidence interval; *STTT*, standard two-tier testing; *MTTT*, modified two-tier testing; *Enz1*, Enzygnost-1; *Enz2*, Enzygnost-2; *Lia*, Liaison; *C6*, C6-ELISA; *RecomL*, RecomLine; *EM*, erythema migrans; *PopC*, population control; *CRC*, cross-reactive control; *strict*, counting equivocal EIA results as negative; *permissive*, counting equivocal EIA results as positive

**Table 3:** Specificity of all algorithms

	Single-tier			STTT			MTTT-strict		MTTT-permissive	
	No. of true negative (specificity %)	95%-CI		No. of true negative (specificity %)	95%-CI		No. of true negative (specificity %)	95%-CI	No. of true negative (specificity %)	95%-CI
**PopC sera (*n*** **= 231)**										
**Enz1**	206 (89.2)^1^	84.4–92.9	**Enz1/RecomL**	224 (97.0)	93.9–98.8	**Enz1/C6**	223 (96.5)^2^	93.3–98.5	221 (95.7)^4,5^	92.2–97.9
**Enz2**	206 (89.2)^1^	84.4–92.9	**Enz2/RecomL**	224 (97.0)	93.9–98.8	**Enz2/C6**	223 (96.5)^2^	93.3–98.5	222 (96.1)^3,4,5^	92.7–98.2
**Lia**	210 (90.9)^1^	86.4–94.3	**Lia/RecomL**	225 (97.4)	94.4–99.0	**Lia/C6**	223 (96.5)^2^	93.3–98.5	220 (95.2)*^,5^	91.6–97.6
**C6**	216 (93.5)^1^	89.5–96.3	**C6/RecomL**	225 (97.4)	94.4–99.0					
**CRC sera (*n*** **= 50)**										
**Enz1**	33 (66.0)^1^	51.2–78.8	**Enz1/RecomL**	45 (90.0)	78.2–96.7	**Enz1/C6**	43 (86.0)^2^	73.3–94.2	41 (82.0)^4,5^	68.6–91.4
**Enz2**	33 (66.0)^1^	51.2–78.8	**Enz2/RecomL**	45 (90.0)	78.2–96.7	**Enz2/C6**	43 (86.0)^2^	73.3–94.2	41 (82.0)^4,5^	68.6–91.4
**Lia**	31 (62.0)^1^	47.2–75.4	**Lia/RecomL**	44 (88.0)	75.7–95.5	**Lia/C6**	43 (86.0)^2^	73.3–94.2	42 (84.0)^4,5^	70.9–92.8
**C6**	39 (78.0)^1^	64.0–88.5	**C6/RecomL**	46 (92.0)	80.0–97.8					

^1^*p* < 0.01 as compared with equivalent STTT (McNemar/exact McNemar)

^2^*p* < 0.05 as compared with single-tier C6-ELISA (one-sample *t* test)

^3^*p* < 0.05 as compared with single-tier C6 (exact McNemar)

^4^*p* < 0.01 as compared with equivalent single-tier (exact McNemar) | (*Lia vs. Lia/C6 (permissive) *p* = 0.021)

^5^Not significant as compared with equivalent MTTT-strict (one-sample *t* test)

A test result was considered to be (true) negative when both the IgM and the IgG component were negative. A test result was considered to be (false) positive when either or both components were positive

*95%-CI*, 95% confidence interval; *STTT*, standard two-tier testing; *MTTT*, modified two-tier testing; *Enz1*, Enzygnost-1; *Enz2*, Enzygnost-2; *Lia*, Liaison; *C6*, C6-ELISA; *RecomL*, RecomLine; *EM*, erythema migrans; *PopC*, population control; *CRC*, cross-reactive control; *strict*, counting equivocal EIA results as negative; *permissive*, counting equivocal EIA results as positive

Sensitivities of STTT protocols were much lower (44–47%). Specificities increased to 97% for PopC sera and 88–92% for CRC sera. No significant differences were seen regarding sensitivity and specificity between the various assays as used in STTT (Tables 2 and 3).

We used MTTT in two variants: classifying equivocal results as negative (“strict”) and classifying equivocal results as positive (“permissive”). The highest sensitivity within the MTTT algorithm was achieved using the permissive variant with the Enzygnost-2 in the first tier, and the C6-ELISA in the second (77.6%). The highest specificity was achieved using the strict variant of the MTTT algorithm, irrespective of the first-tier EIA that was used (96.5%). In both variants, the sensitivity of the combination Enzygnost-2/C6 was better than that of the combination Liaison/C6 (strict *p* = 0.003; permissive *p* = 0.007). Other comparisons *within* each algorithm/variant yielded no significant differences (Tables 2 and 3).

When comparing the single-tier, MTTT-strict, or MTTT-permissive algorithms to STTT, we found sensitivity of all these algorithms to be significantly better than the equivalent STTT algorithm. The specificity of all assays in the single-tier algorithm was significantly lower than that of STTT. However, the specificity of both MTTT strategies in the population controls and in controls with a potentially cross-reactive condition was comparable with that of STTT.

Comparing the permissive variant of the MTTT algorithm to the strict variant, we found that the permissive strategy resulted in a higher sensitivity for all assays (*p* < 0.01). This increase in sensitivity was 4.8% (95% CI 2.0–7.6) for the Enzygnost-1/C6 and Enzygnost-2/C6, and 5.7% (95% CI 2.6–8.7) for the Liaison/C6. Interestingly, specificity in both population controls and controls with a potentially cross-reactive illness was comparable between the two variants. The MTTT-permissive algorithm also outperformed equivalent single-tier assays, as all MTTT-permissive combinations had comparable sensitivity, but a higher specificity than their single-tier equivalent.

Comparing all MTTT combinations to the “benchmark” C6-ELISA, we found unsurprisingly that the assays in the strict variant had a significantly better specificity than the single-tier C6-ELISA. These combinations did all have significantly lower sensitivity. As previously mentioned, all MTTT-permissive variants had a better sensitivity than their equivalent strict variant, and two of such combinations (Enz1/C6 and Enz2/C6) even had comparable sensitivity with the single-tier C6-ELISA. Importantly, for the best scoring combination (Enz2/C6), this did not come at the cost of a loss of specificity, which was still better than that of the single-tier C6-ELISA (specificity PopC C6 93.5% vs. C6/Enz2 96.1%, *p* = 0.03). All comparisons between MTTT variants and the C6-ELISA are given in Tables 2 and 3.

The diagnostics odds ratios for the various assays and algorithms are given in Table 4.

**Table 4:** Diagnostic odds ratios of all algorithms

	Single-tier DOR (95%-CI)		STTT DOR (95%-CI)		MTTT-strict DOR (95%-CI)	MTTT-permissive DOR (95%-CI)
DOR with PopC sera						
**Enz1**	30.9 (18.1–52.1)	**Enz1/RecomL**	27.8 (12.5–61.6)	**Enz1/C6**	68.4 (32.0–146.5)	69.5 (34.4–140.3)
**Enz2**	34.5 (20.3–58.4)	**Enz2/RecomL**	27.8 (12.5–61.6)	**Enz2/C6**	74.6 (34.8–160.1)	85.6 (41.0–178.6)
**Lia**	23.5 (13.8–40.0)	**Lia/RecomL**	29.8 (12.7–69.9)	**Lia/C6**	56.9 (26.6–121.3)	53.6 (27.3–104.9)
**C6**	51.3 (27.9–94.4)	**C6/RecomL**	30.3 (12.9–71.2)			
DOR with CRC sera						
**Enz1**	7.3 (3.7–14.2)	**Enz1/RecomL**	7.8 (2.9–20.4)	**Enz1/C6**	15.1 (6.5–35.2)	14.3 (6.5–31.3)
**Enz2**	8.1 (4.2–15.9)	**Enz2/RecomL**	7.8 (2.9–20.4)	**Enz2/C6**	16.5 (7.0–38.5)	15.8 (7.2–34.6)
**Lia**	3.8 (2.0–7.3)	**Lia/RecomL**	5.8 (2.4–14.2)	**Lia/C6**	12.5 (5.4–29.2)	14.0 (6.2–31.6)
**C6**	21.6 (6.0–26.4)	**C6/RecomL**	9.3 (3.2–26.7)			

*DOR*, diagnostic odds ratio; *95%-CI*, 95% confidence interval; *STTT*, standard two-tier testing; *MTTT*, modified two-tier testing; *Enz1*, Enzygnost-1; *Enz2*, Enzygnost-2; *Lia*, Liaison; *C6*, C6-ELISA; *RecomL*, RecomLine; *EM*, erythema migrans; *PopC*, population control; *CRC*, cross-reactive control; *strict*, counting equivocal EIA results as negative; *permissive*, counting equivocal EIA results as positive

**Fig. 2:**
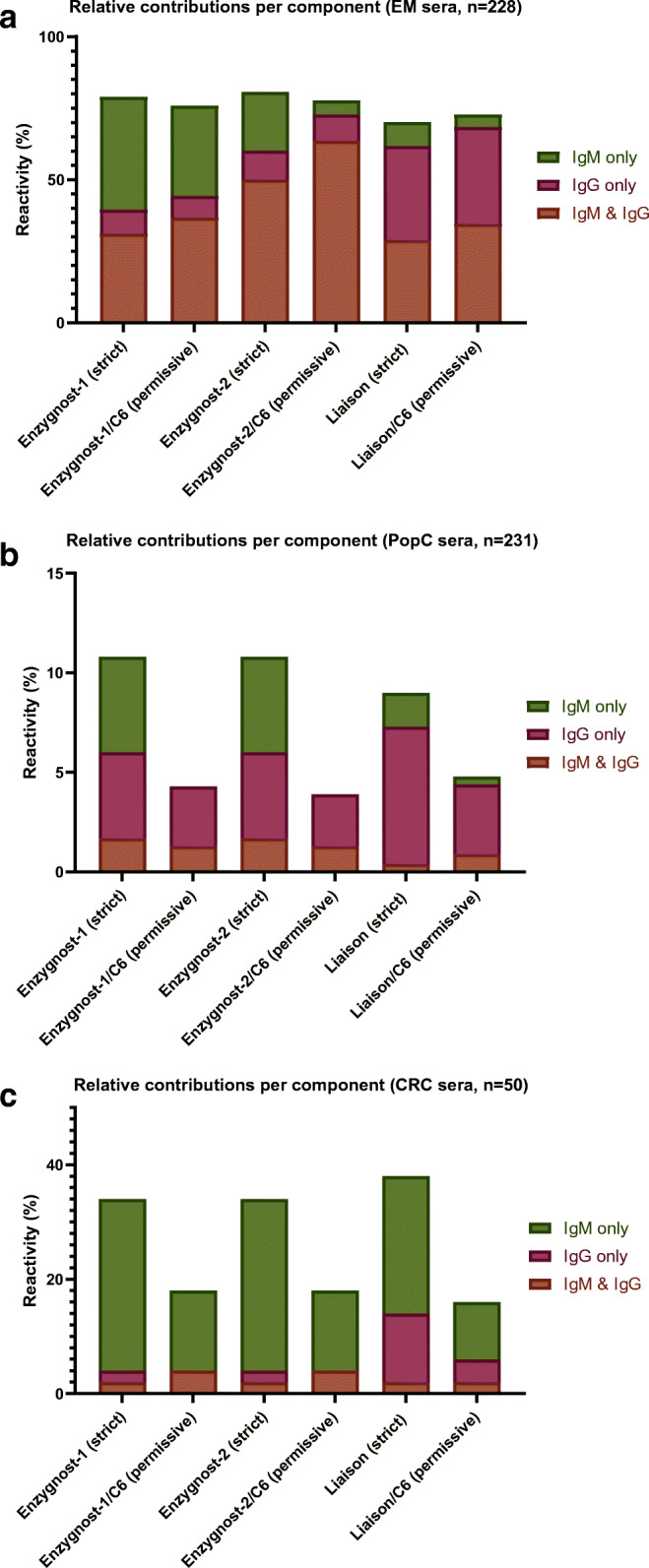
Reactivity per component - single-tier & MTTT-permissive (C6 and RecomL not shown) **a** EM sera, percentages calculated from n=228. **b** PopC sera, percentages calculated from n=231. **c** CRC sera, percentages calculated from n=50

Among the screening EIAs, the relative contribution of the IgM component was highest in the Enzygnost-1 assay. That assay’s IgG component, which does not contain VlsE, performed rather poorly (Fig. 2a, orange and pink bars combined), as did the IgM component of the Liaison, which relies on recombinant antigens only (Fig. 2a, orange and green bars combined). Use of MTTT was most useful for correct classification of IgM-positive PopC sera (Fig. 2b) and also filtered out a significant number of IgM false-positive CRC sera (Fig. 2c). Based on Fig. 2a–c, it could be hypothesized that using only the IgG component of each first-tier assay would result in a large improvement of specificity in CRC sera with only a minor decrease in sensitivity. Diagnostic parameters of this MTTT-permissive IgG-only variant are given in Table 5. This variant without a separate IgM component had the expected increase in specificity in CRC sera, but this did come at the cost of a statistically significant decrease in sensitivity for all combinations. Within the IgG-only variant, the Enz2-IgG/C6 outperformed both other combinations (both comparisons *p* < 0.01). Further subanalysis of both MTTT algorithms showed that Enzygnost-2-IgG outperformed Liaison-IgG in terms of sensitivity (*p* = 0.041), while having comparable specificity in PopC and CRC sera, *but only* in the permissive variant (strict variant, sensitivity Enz1-IgG vs. Lia-IgG *p* = 0.664) (Supplementary Table 1).

**Table 5:** Diagnostic parameters of algorithms with IgG component only

**EM sera (*n*** **= 228)**					
	**MTTT-permissive**			**MTTT-permissive IgG-only**	
	No. of true positive (sensitivity %)	95%-CI		No. of true positive (sensitivity %)	95%-CI
**Enz1/C6**	173 (75.9)	69.8–81.3	**Enz1-IgG/C6**	101 (44.3)*	37.7–51.0
**Enz2/C6**	177 (77.6)	71.7–82.9	**Enz2-IgG/C6**	166 (72.8)*	66.5–78.5
**Lia/C6**	166 (72.8)	66.5–78.5	**Lia-IgG/C6**	156 (68.4)*	62.0–74.4
**PopC sera (*n*** **= 231)**					
	**MTTT-permissive**			**MTTT-permissive IgG-only**	
	No. of true negative (specificity %)	95%-CI		No. of true negative (specificity %)	95%-CI
**Enz1/C6**	221 (95.7)	92.2–97.9	**Enz1-IgG/C6**	221 (95.7)	92.2–97.9
**Enz2/C6**	222 (96.1)	92.7–98.2	**Enz2-IgG/C6**	222 (96.1)	92.7–98.2
**Lia/C6**	220 (95.2)	91.6–97.6	**Lia-IgG/C6**	221 (95.7)	92.2–97.9
**CRC sera (*n*** **= 50)**					
	**MTTT-permissive**			**MTTT-permissive IgG-only**	
	No. of true negative (specificity %)	95%-CI		No. of true negative (specificity %)	95%-CI
**Enz1/C6**	41 (82.0)	68.6–91.4	**Enz1-IgG/C6**	48 (96.0)*	86.3–99.5
**Enz2/C6**	41 (82.0)	68.6–91.4	**Enz2-IgG/C6**	48 (96.0)*	86.3–99.5
**Lia/C6**	42 (84.0)	70.9–92.8	**Lia-IgG/C6**	47 (94.0)*	83.5–98.8

*p < 0.05 as compared with equivalent MTTT-permissive (one-sample *t* test)

*95%-CI*, 95% confidence interval; *MTTT*, modified two-tier testing; *Enz1*, Enzygnost-1; *Enz2*, Enzygnost-2; *Lia*, Liaison; *C6*, C6-ELISA; *EM*, erythema migrans; *PopC*, population control; *CRC*, cross-reactive control; *permissive*, counting equivocal EIA results as positive; *IgG only*, using only the IgG component of the first-tier assay

Subanalysis of IgG-blot positive vs. IgG-blot negative EM sera showed that the additional value of VlsE in the IgG-EIAs was restricted to blot-negative sera, i.e. early infection (IgG-blot NEG, Enz2-IgG/Lia-IgG vs. Enz1-IgG *p* < 0.01; IgG-blot POS, all comparisons *p* > 0.05) (Supplementary Table 2).

## Discussion

In our study, we have evaluated various algorithms of single-tier testing or MTTT compared with STTT in European patients with early localized LB. Previous studies conducted in the USA have shown that MTTT protocols generally improve sensitivity of serological tests for LB without losing specificity [7, 13–15]. Our results show that the same holds true for the European situation, even when the screening EIA contains VlsE and the confirmatory test is the C6-ELISA, which functions with the IR6 peptide of VlsE.

Overall, the best diagnostic parameters were found using the Enzygnost-2 in the first tier, even though differences with the other assays within any algorithm were mostly non-significant. The highest sensitivity was found when using this assay as a standalone single-tier test. As was to be expected, this did result in a markedly lower specificity. Conversely, STTT had the best specificity, but lacked sensitivity in the EM sera that were used. The sensitivity of STTT in our study was comparable with the reported sensitivity of serological testing in EM patients in a meta-analysis by Leeflang and colleagues [11], and we therefore consider it to be representative of EM patients in general.

The algorithms of MTTT that we studied all showed marked improvement of sensitivity as compared with STTT, without significant loss of specificity. Because MTTT is quite new in Europe, we also investigated which “rules” should be used for assay interpretation. Interestingly, we found that classifying equivocal results as positive resulted in an increase of sensitivity and only a negligible and non-significant decrease in specificity. For each assay, this permissive approach maintained comparable sensitivity with their single-tier equivalents, but with a significantly better specificity.

Finally, it must be noted that the best performing combination (Enzynost-2/C6 in the permissive variant) had a comparable sensitivity with the “benchmark” C6-ELISA, but a significantly better specificity in population controls. While the absolute differences were small (C6 93.5% vs. Enz2/C6 96.1%), this is still of great significance in the field of LB diagnostics. *Borrelia* serology is frequently requested for patients with a low pre-test probability of having LB; hence, the impact of a small improvement in specificity on the eventual positive predictive value can be substantial. This implies that two-tier testing is still advantageous, in spite of the C6-ELISA’s excellent diagnostic parameters. It must be noted that further improvements to the diagnostic parameters of multi-tier *Borrelia* serology may be possible when using more innovative ways of combining assays or assays’ components than the simple Boolean logic (i.e. AND/OR) we have employed in the current study [19, 20]. Our findings do not support dropping the IgM component from the first-tier assays, even in assays which have VlsE in their IgG component, even though such a move has recently been gaining popularity [21, 22].

One might argue that serological testing for an EM is not good clinical practice. After all, clinical guidelines state that an EM is considered a clinical diagnosis [1–3]. However, this is in part because current serological algorithms lack adequate sensitivity. Taking into account our findings, it may be prudent to further investigate the applicability of MTTT for patients with early LB. Of course, treatment of a cutaneous lesion which is recognized as an EM should not be delayed by serological confirmation, as antibodies may not always have formed. However, both our and previous studies show that requests for serology are nonetheless frequent in the setting of Dutch general practice, even in patients with clear-cut EM [8]. This implies that patients and clinicians value the added certainty that laboratory work-up gives to the physician’s clinical assessment. Laboratory testing may be especially relevant in situations where the LB diagnosis is not clear-cut, for example, when skin lesions do not resemble a typical EM, or if patients present with only generalized symptoms without a skin lesion [23, 24]. Considering the improved diagnostic parameters of MTTT, serology may serve to aid in the diagnosis of these situations. All of the aforementioned does not negate the recommendation, however, that cutaneous lesions should be treated as an EM without awaiting serological outcomes, if the clinician recognizes them as such. Finally, it is important to note once more that the true value of any MTTT algorithm will depend on the setting in which the algorithm is used (i.e. the pre-test probability and resulting positive and negative predictive values).

Our study also indicates that the IgM and IgG components of the different assays rely on different antigens for their reactivity. The IgG assay without VlsE was substantially less reactive in EM sera, as was the IgM assay which did not contain a whole cell extract but relied on recombinant antigens only. This last finding is in agreement with a recent study from Northern Europe [25]. Our subanalysis allows for a tentative hypothesis that the VlsE from the Enzygnost-2, consisting of three species, performs better than Liaison’s Bbav-VlsE; however, these results depended on the algorithm used (strict vs. permissive). Further studies should confirm whether addition of VlsE from different species improves diagnostic accuracy.

A limitation of our study is that we did not include a polymerase chain reaction (PCR) or culture on skin biopsies obtained from the EM to verify the presence of Bbsl in these lesions. However, the sensitivity of culture or PCR is not perfect [26, 27]. Excluding EMs in which the spirochete cannot be directly detected may therefore lead to an overestimation of the sensitivity. Branda and colleagues [13] also found a trend towards higher sensitivity of their MTTT algorithms in patients with culture-confirmed EM compared with culture-negative EMs. Our study might also have benefitted from multiple sera from the same patient from both the acute and convalescent phase; however, these were not available for a sufficient number of patients in this convenience sample. In contrast, a strength of our study was that the population controls were drawn from a representative sample of the population as a whole, whereas many studies use blood bank donors as controls. Blood bank donors are inherently a healthier subset of the entire population, with the potential for bias towards a different background seroprevalence.

Due to the rarity of definite and probable disseminated LB, we could not collect sufficient sera during the study period to include these in the study as well. Even though one of the major advantages of MTTT (i.e. improved sensitivity) primarily applies to early (localized) LB, it is imperative that future studies compare different MTTT algorithms for patients with disseminated LB as well. Even though the expected improvement in diagnostic parameters will likely be smaller, other advantages of MTTT will still apply: EIAs require less labor and hence lower costs of LB serology, and due to their quantitative nature have a decreased risk of inter-assay variation [28].

As a final remark, it is evident that MTTT protocols are not the solution to some of the other inherent problems of serology for LB diagnostics. MTTT does not solve the problem of distinguishing between an active and past infection after IgG-seroconversion, and all serology still depends on indirect methods of detection, as opposed to direct detection of the causative microorganism. MTTT protocols may, however, be a partial solution to some of the problems surrounding serology, which cause controversy among doctors and patients.

## Electronic supplementary material

ESM 1 (PDF 207 kb).

ESM 2 (DOCX 32 kb).

## Data Availability

The dataset generated during and/or analyzed during the current study is available from the corresponding author on reasonable request.
