# Supplementary material for: Diagnostic parameters of modified two-tier testing in European patients with early Lyme disease
Source: Eur J Clin Microbiol Infect Dis. 2020 Jul 6;39(11):2143–52. doi: 10.1007/s10096-020-03946-0 (PMC7561539; doi:10.1007/s10096-020-03946-0)
Supplement: Supplementary file 1 — (PDF 207 kb). [file 10096_2020_3946_MOESM1_ESM.pdf]

SUPPLEMENTARY DATA
European Journal of Clinical Microbiology & Infectious Diseases

Modified two-tier testing in European patients with early Lyme disease

M.E. (Ewoud) Baarsma [1], J.F.P. (Joop) Schellekens [2,3], B.C. (Bart) Meijer [2], A.H. (Afke) Brandenburg [4], Thorhold Souilljee [2], Agnetha Hofhuis [5], J.W. (Joppe) Hovius [1], A.P. (Alje) van Dam [6,7]

Affiliations

1. Amsterdam UMC, University of Amsterdam, Center for Experimental and Molecular Medicine, Amsterdam Infection & Immunity Institute, Amsterdam, the Netherlands
2. Certe Laboratory of Infectious Diseases, Groningen, the Netherlands
3. Centre for Infectious Diseases Research Diagnostics and Laboratory Surveillance, National Institute of Public Health and the Environment (RIVM), Bilthoven, the Netherlands
4. Izore, Centrum Infectieziekten Friesland, Leeuwarden, the Netherlands
5. Epidemiology and Surveillance unit, Centre for Infectious Disease Control, National Institute of Public Health and the Environment (RIVM), Bilthoven, the Netherlands
6. Department of Medical Microbiology, OLVG, , Amsterdam, the Netherlands
7. Amsterdam UMC, University of Amsterdam, Department of Medical Microbiology, Amsterdam, the Netherlands

Corresponding author

M.E. (Ewoud) Baarsma, MD
Meibergdreef 9
1105 AZ Amsterdam, the Netherlands
e: lyme@amc.nl | ORCID: 0000-0001-8090-3602

SUPPLEMENTARY TABLE 1
comparison of reactivity of Enzygnost-VlsE vs. Liaison-VlsE

		EM sera		PopC sera		CRC sera	
		No. true-positive / n	p-value	No. false-positive / n	p-value	No. false-positive / n	p-value
**MTTT – strict**	**Reactive with Enz2-IgG/C6 only** (Enz2-IgG POS & Lia-IgG NEG)	9 / 228	0.664	1 / 231	1.000	0 / 50	1.000
	**Reactive with Lia-IgG/C6 only** (Enz2-IgG NEG & Lia-IgG POS)	12 / 228		1 / 231		0 / 50	
	**Reactive with Enz-VlsE/C6 only** (Enz1-IgG NEG & Enz2-IgG POS & Lia-IgG NEG)	6 / 138	0.332	0 / 217	1.000	0 / 48	1.000
	**Reactive with Lia-VlsE/C6 only** (Enz1-IgG NEG & Enz2-IgG NEG & Lia-IgG POS)	11 / 138		1 / 217		0 / 48	
		EM sera		PopC sera		CRC sera	
		No. true-positive / n	p-value	No. false-positive	p-value	No. false-positive	p-value
**MTTT – permissive**	**Reactive with Enz2-IgG/C6 only** (Enz2-IgG POS & Lia-IgG NEG)	15 / 228	0.041	1 / 231	1.000	0 / 50	1.000
	**Reactive with Lia-IgG/C6 only** (Enz2-IgG NEG & Lia-IgG POS)	5 / 228		2 / 231		1 / 50	
	**Reactive with Enz-VlsE/C6 only** (Enz1-IgG NEG & Enz2-IgG POS & Lia-IgG NEG)	10 / 127	0.180	0 / 209	0.500	0/ 46	1.000
	**Reactive with Lia-VlsE/C6 only** (Enz1-IgG NEG & Enz2-IgG NEG & Lia-IgG POS)	4 / 127		2 / 209		1 / 46	

p-values were calculated using (exact) McNemar test

Comparisons are shown including Enz1-IgG EQUI/POS sera (upper two rows for both strict and permissive algoritms) and excluding them (lower two rows). Excluding sera that are Enz1-IgG POS (strict) or Enz1-IgG EQUI/POS (permissive) enables a tentative comparison of sera which were Enz2-VlsE reactive *only* (i.e. not reactive to WCS) with sera that were Lia-VlsE reactive only. Samples that were reactive to both Enz2-VlsE and Lia-VlsE, or to neither, are not counted in the comparison.

SUPPLEMENTARY TABLE 2:
reactivity of IgG-EIAs in IgG-blot negative vs. IgG-blot positive EM sera (n=207)

	**Single-tier**					**MTTT – permissive**			
	**IgG-blot POS (n=46)**		**IgG-blot NEG (n=161)**			**IgG-blot POS (n=46)**		**IgG-blot NEG (n=161)**	
	No. reactive (%)	95%-CI	No. reactive (%)	95%-CI		No. reactive (%)	95%-CI	No. reactive (%)	95%-CI
**Enz1-IgG**	44 (95.7)	85.2-99.5	46 (28.6)	21.7-36.2	**Enz1-IgG/C6**	45 (98.8)	88.5-99.9	56 (34.8)	27.5-42.7
**Enz2-IgG**	46 (100)	92.2-100	91 (56.5)	48.5-64.3	**Enz2-IgG/C6**	46 (100)	92.2-100	120 (74.5)	67.1-81.1
**Lia-IgG**	44 (95.7)	85.2-99.5	97 (60.2)	52.3-67.9	**Lia-IgG/C6**	44 (97.7)	85.2-99.5	112 (69.6)	61.8-76.6

Only single-tier and MTTT-permissive algorithms are shown. EM sera for which no immunoblot was performed were excluded (n=21).
For some cells, reactivity in the MTTT-permissive algorithms exceeds that of the equivalent (strict) single-tier algorithm due to classification of equivocal results as positive.

Abbreviations/explanations: 95%-CI: 95% confidence interval; Enz1: Enzygnost-1; Enz2: Enzygnost-2; Lia: Liaison; C6: C6-ELISA; EM: erythema migrans; MTTT: modified two-tier testing; permissive: counting equivocal EIA results as positive.
